# Isolation of Thermophilic Bacteria and Investigation of Their Microplastic Degradation Ability Using Polyethylene Polymers

**DOI:** 10.3390/microorganisms10122441

**Published:** 2022-12-09

**Authors:** Sadin Özdemir, Ceyhun Akarsu, Ömer Acer, Mireille Fouillaud, Laurent Dufossé, Nadir Dizge

**Affiliations:** 1 Food Processing Programme, Technical Science Vocational School, Mersin University, Mersin 33343, Turkey; 2 Department of Environmental Engineering, Istanbul University-Cerrahpasa, Istanbul 34320, Turkey; 3 Department of Medical Microbiology, Medical Faculty, Siirt University, Siirt 56100, Turkey; 4 Chemistry and Biotechnology of Natural Products, CHEMBIOPRO, ESIROI Agroalimentaire, Université de La Réunion, 15 Avenue René Cassin, CS 92003, CEDEX 9, F-97744 Saint-Denis, France; 5 Department of Environmental Engineering, Mersin University, Mersin 33343, Turkey

**Keywords:** biodegradation, fourier transform infrared spectroscopy, microplastic degradation, polyethylene, thermophilic bacteria

## Abstract

Microplastics (MPs) pose potential public health challenges because of their widespread occurrences in all environmental compartments. While most studies have focused on the occurrence fate of microplastics in wastewater treatment systems, the biodegradation of microplastics in wastewater is generally little understood. Therefore, we used two Gram-positive and thermophilic bacteria, called strain ST3 and ST6, which were identified by morphological, biochemical, physiological, and molecular analyses, to assess the growth and biodegradation potential of two different sized (50 and 150 m) polyethylene particles. The degradation was monitored based on structural and surface morphological changes. According to 16S rRNA analyses, ST3 and ST6 were identified as *Anoxybacillus flavithermus* ST3 and *Anoxybacillus* sp. ST6, respectively. The occurrence of cracks, holes, and dimensional changes was detected by scanning electron microscopy. Moreover, critical characteristic absorption band formation and modifications were determined by Fourier transform infrared spectroscopy. In addition to these, it was found that *Anoxybacillus flavithermus* ST3 and *Anoxybacillus* sp. ST6 produced high level of alpha-Amylase. These results showed that thermophilic bacteria are capable of the biodegradation of microplastics and production of alpha-Amylase.

## 1. Introduction

Human activities, which are significant components of global pollution, are the largest source of many pollutants, including plastics, due to broad usage and inappropriate disposal [1,2]. Plastic usage has been steadily increasing over the last 50 years as a result of technological improvements [3]. In fact, assuming current growth rates hold, by 2050, the world’s plastic manufacturing will have tripled from 350 million tons to 1100 tons [4]. However, the rate of inadequately managed waste has been estimated at a mean of 50% [5,6]. As a result, a significant amount of plastic may build in vast amounts in rivers, waterways, and coastal regions, leading to the creation of microplastics [7,8].

Microplastics, which are plastics less than 5 mm, are divided into different groups as primary and secondary types, sizes, shapes, and chemical properties [9,10,11]. Mostly, secondary microplastics have the same chemical properties whatever polymer the plastic is made of.

Many sectors have been related to the occurrence of microplastics in freshwater [12], marine [13], and wastewater treatment plants [14], including textiles, laundry services, and raw plastics manufacturing. According to numerous studies published in the last few years, the primary source of microplastics reaching the environment is wastewater treatment plants [15,16]. Therefore, some studies focused on determining and increasing microplastic removal of wastewater treatment plants to prevent microplastic pollution in marine ecosystems. However, these current methods practically cause a trade-off between water and sludge soil [17]. Moreover, the removal of the potential of the WWTPs is uncertain hence they are not currently built up to remove microplastics [18]. Even if WWTP removes up to 95% of microplastics, the remaining 5% is still a significant amount on account of the high volume of generated wastewater effluent [19]. Carr et al. (2016) reported that 9.3 × 10^5^ MP is discharged from the WWTP each day even with 99% removal efficiency [20]. With an average removal rate, WWTPs can emit ranging from 4.2 × 10^4^ and 13.9 × 10^10^ MPs/day [21]. It should be noted that sewage sludge contains the majority of the retained microplastics, which range from 1.0 to 24.0 MP per gram [22,23]. Therefore, it’s critical to create a process that can turn this pollutant into a finished good.

**Figure 1:**
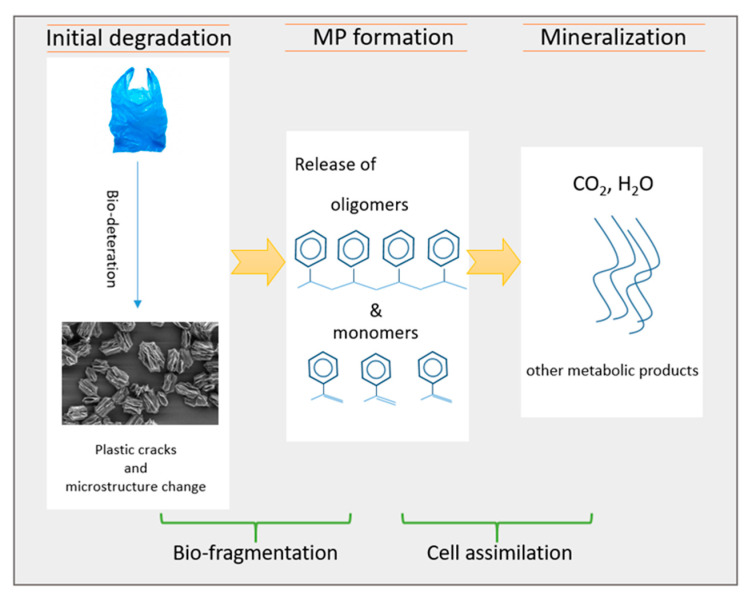
Overview of the degradation of conventional plastic waste.

In the degradation of plastics and MPs, the polymer molecules in such plastics first begin to degrade by organisms (Figure 1). Then the polymers turn into polymer fragments, and the carbon in the polymer chain, which is finally degraded and undergoes mineral degradation. Thus, polymer degradation by microorganisms takes place [24,25]. The primary factor affecting the biodegradation of microplastics is a polymer’s characteristics, which include its chemical composition, molecular mass, and degrees of crystallinity [26]. Besides, appropriate enzymatic and metabolic pathways must be possessed to enhance biodegradation by potential microbial degrading organisms.

So far, more than 20 bacterial genera including various Gram-negative and Gram-positive species have been successfully isolated from the natural environment such as sludge, sediment, and wastewater [27], and the degradation of polyethylene has been investigated [28]. Additionally, the complete biodegradability of polyethylene was enhanced by adding several additives. For example, Arkatkar et al. (2010) reported the complete degradation of PE particles with *P. fluorescens* in presence of surfactant and bio-surfactant [29]. Similarly, Tribedi and Sil (2013) also reported that the addition of mineral oil has slightly increased the degradation of LDPE films [30].

However, only a few of them have investigated the biodegradation of microplastics with a limited number of polymer types [31,32]. Current studies on bacterial isolates, their degradation properties, and their effects on MPs are given in Table 1. Auta et al. (2017) carried out one of the first studies on microplastic degradation after incubation with *Rhodococcus* sp. strain 36 and *Bacillus* sp. strain 27 [33]. Accordingly, the weight loss were 6.4% and 4.0% for *Rhodococcus* sp. strain 36 and *Bacillus* sp. strain 27, respectively. Further studies demonstrate that bacteria can predominantly change the appearance of MPs including their chemical and other properties. Thermophilic bacteria, for example, may live in a range of conditions and generate oxidation and hydrolase enzymes, presenting a significant possibility for microplastic biodegradation resulting in numerous cracks and grooves [34,35]. The maximum weight loss of polymer particles was reached with *Bacillus* strains, which indicates that this species has a relatively better degradation effect on plastic particles [27,36].

In this study, novel amylase-producing thermophilic bacteria were used to examine the possible degradation of non-biodegradable MPs. The amylase enzyme is known to belong to the hydrolase family, which can hydrolytically degrade long polymer chains into smaller monomers including polymers [46,47]. Specifically, many studies deal with the enzymatic degradation of polymers with particular attention to investigating the influence of processing conditions, types of enzymes used for degradation, and processing times [48,49]. Consequently, the potential of the biodegradability of MPs using the thermophilic *Anoxybacillus* genus was investigated. Moreover, scanning electron microscopy (SEM) was used to investigate the morphological changes on microplastic surfaces. Changes in polymer functional groups were analyzed using Fourier-transform infrared spectroscopy (FTIR).

## 2. Materials and Methods

### 2.1. Sampling, Pre-Enrichment, and Characterization

The water and muddy soil samples from Ömer hot springs were used to isolate thermophilic bacteria in Afyonkarahisar, Turkey. An enrichment medium by serial dilution approach was utilized to isolate thermophilic bacteria. For this, nutritional broth (NB, agar 1.5%) was used to culture a suspension of soil and water samples for 24 h at 55 °C. After 1–2 cycles of enrichment, the cultures were plated for 48 h on a solid NB medium. The colonies with varied phenotypes on the plates were then moved to a new fresh medium. Strong amylolytic bacteria were selected by adding Lugol’s iodine solution after incubation of the bacteria on starch agar plates as previously described by Acer et al. (2020) [50].

Gram staining was carried out according to Dussault (1955) [51]. Growing bacteria at different temperatures and pH ranges were used to evaluate the impact of temperature and pH on growth. The novel isolates were cultivated in a liquid Basal Salt Medium (BSM) to investigate the use of a single carbon source. A final concentration of 10 g/L was achieved by adding carbon sources as filter-sterilized solutions. All growth assays were conducted at 55 °C and a pH of 6.0. Carbon sources included glucose, lactose, and sucrose. Following a two-day incubation period, positive growth was seen when the OD values at 600 nm were more than 0.300. The oxidase activity was determined by oxidation of p-aminodimethylaniline oxalate. Hydrogen peroxide solution (3% *v*/*v*) was used to assess catalase activity. The urease activity was determined as described by Lanyi (1988) [52]. Citrate utilization was determined by using Simmons’ citrate agar and indole production was determined by Kovac’s reagent. Through the use of an NB medium with various concentrations of NaCl, the tolerance to NaCl was evaluated. To evaluate β-galactosidase activity, the release of o-nitrophenyl-D-galactopyranoside (o-NPG, Sigma) was quantified. The phosphatase activity was investigated performing agar medium containing both phenolphthalein diphosphate and methyl green; in these culture media, phosphatase-producing microorganisms grow deep-green-stained colonies, whereas non-producing microorganisms do not.

### 2.2. Phylogenetic Analysis and Bioinformatics Processing

DNA isolation of the samples was realized as previously mentioned by Akarsu et al. (2022) [53]. For identification of the species, partial 16S rRNA genes were amplified in PCR analysis using universal primers (PA5′AGAGTTTGATCCTGGCTCAG-3′-PH5′ AAGGAGGTGATCCAGCCGCA-3′) [54], which amplify the complete 16S rRNA Gene Iontek Company (Istanbul, Turkey) used an AB1373 Automated Sequencer from Invitrogen in Carlsbad, California to sequence the 16S rRNA gene.

The raw sequence data were pre-processed as previously described [53]. To get rid of blank spaces and incomplete data, the sequences were cleaned up. Partial 16S rRNA gene sequences of the strains were used to perform homology analysis. We compared the sequences to those in the National Center for Biotechnology Information (NCBI) database using the Basic Local Alignment Search Tool (BLAST). After CLUSTAL X performed multiple alignments on the data, we used Molecular Evolutionary Genetic Analysis (MEGA) version X to construct the phylogenetic sequence tree [55]. A distance based method called “The Neighbor-Joining” was used to infer the evolutionary history [56]. The evolutionary distance was measured by the Maximum Composite Likelihood method [57].

### 2.3. Biodegradation Assay and Analysis

Polyethylene is chemically inert and is regarded as the most difficult polymer to recycle [58]. Accordingly, the main polymer composition of MPs was polyethylene in wastewater treatment plants [59]. Despite the fact that particle size is an important independent factor influencing the rate of surface erosion as well as polymer type [60], very few studies have evaluated the effect of particle diameter [53,61]. Therefore, two different-sized polyethylene microplastics (PE-MPs) were used in this study.

Polyethylene particles known as Aldrich-434272 and Goodfellow-9002884 have a size of 50 µm and 150 µm, respectively. In order to counteract photo-degradation, the degradation experiment was conducted for 10 days without light. After the period, the remaining polyethylene particles were filtered for further analysis.

The microbial biodegradation studies were carried out in 250 mL Erlenmeyer flasks containing 100 mL using Nutrient Broth medium. After sterilization, 20 mL of the sample was taken from the original culture and transferred into tube, and then cultured at 140 rpm/min on a shaker, and periodically sampled and tested.

**Figure 2:**
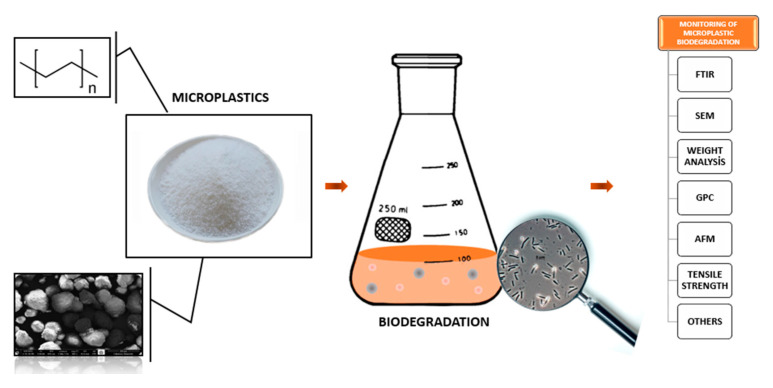
Microplastic biodegradation detection methods in the laboratory.

So far, several techniques such as FTIR, GC-MS, SEM, tensile strength, and weighting had been used to reveal biodegradation [62] (Figure 2). In the present study, the changes in PE MPs’ structure were analyzed by FTIR Spectroscopy (PerkinElmer, Inc., Waltham, MA, USA) in the frequency range of 4000–400 cm^−1^. SEM (Gemini Zeiss Supra 55, Oberkochen, Germany) was used to view the morphology of the degraded PE MPs’. In addition, the carbonyl index was used to evaluate the oxidation states of MPs and the deterioration in their mechanical properties in this study [63]. The carbonyl index has a fixed location and is free from other bands that may interfere since it has been used for many years to monitor changes in the carbonyl band (C = O) [64].

(1) $$Carbonyl\;index=\frac{A_{1}}{A_{2}}$$

where $A_{1}$ is the absorbance at 1720 cm^−1^ and $A_{2}$ is the reference peak of polyethylene [63].

## 3. Results

### 3.1. Characterization of Phenotypic Diversity

In the present study, two thermophilic bacterial species, which are Gram-positive, rod-shaped, spore-forming, and motile, able to hydrolyze starch, named ST3 and ST6 strains, respectively, were isolated from habitats in Ömer thermal springs (Turkey) (Table 2).

**Figure 3:**
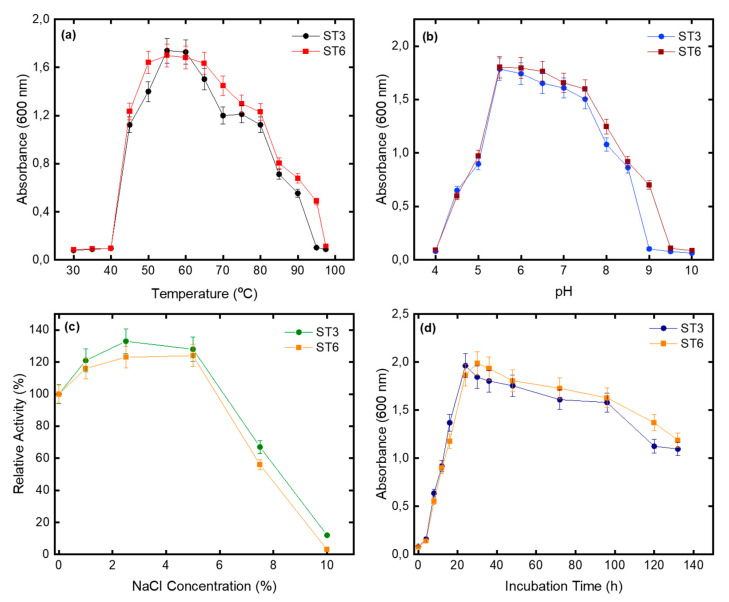
Effect of (**a**) temperature, (**b**) pH, (**c**) NaCI on microbial growth, and (**d**) microbial growth kinetics.

β-galactosidase, amylase, catalase, oxidase, citrate, and phosphate tests were all positive for strain ST3; however, urease and protease were negative. As carbon sources, the strain could use glucose, lactose, and sucrose. As shown in Figure 3a,b, 55–60 °C and a pH of 5.5–6.0 were the ideal media for microbial growing, respectively. It was determined that if the NaCl concentration was 1–7.5% (*w*/*v*), it did not prevent the growth of ST3 strain and ST6 strain, and accordingly, values between 2.5–5% (*w*/*v*) and 1–5% (*w*/*v*) were considered suitable for the optimum concentration, respectively (Figure 3c). Amylase, catalase, oxidase, citrate, phosphate, and protease tests for strain ST6 were found to be positive, and it was discovered that these enzymes could degrade glucose, lactose, and sucrose as carbon sources. The growth kinetics of both bacteria under optimum conditions are also presented in Figure 3d.

**Figure 4:**
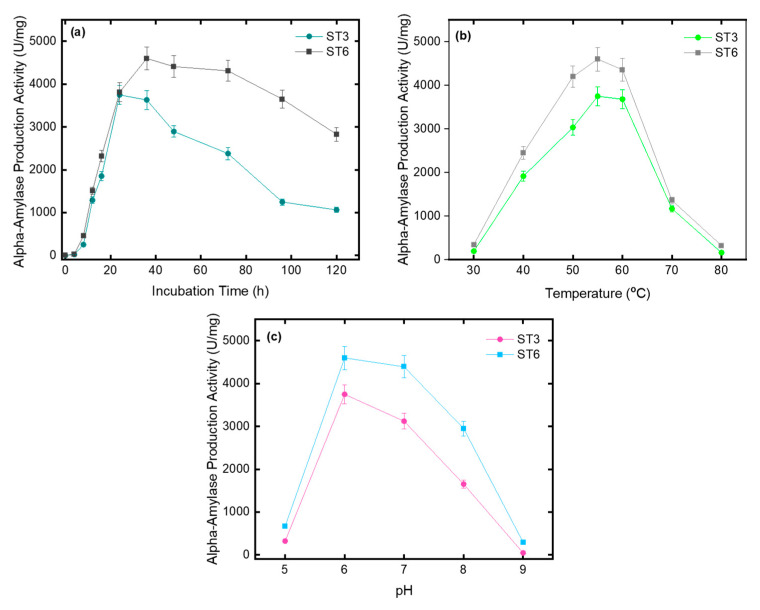
Effect of (**a**) incubation time, (**b**) temperature, (**c**) pH on alpha-amylase production.

The effect of incubation time on amylase production of ST3 and ST6 was tested and the results are shown in Figure 4a. As can be clearly seen in Figure 4a, both ST3 and ST6 were found good sources of alpha amylase. Amylase production increased rapidly from the 12th h and reached the 24th h maximum level for strain ST3 and also 36th h maximum level for strain ST6. ST3 and ST6 exhibited near-maximum amylase production activity between 24–36th and 36–72nd h. In addition to these, the effect of incubation temperature (Figure 4b) and fermentation pH (Figure 4c) on alpha-amylase production were examined. It was also determined that the optimum incubation temperature and fermentation pH was found as 55 °C and 6.0 for ST3 and ST6.

### 3.2. Phylogenetic Analyses

**Figure 5:**
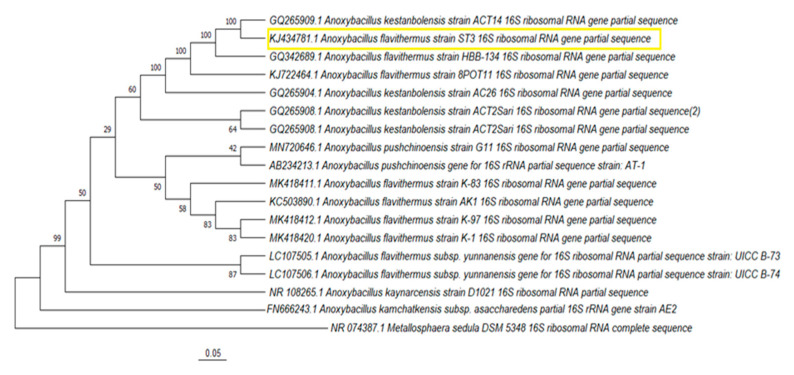
Phylogenetic tree depicting the relationships between strain ST3 and related taxa. The used outgroup was *Metallosphaera sedula* DSM 5348.

**Figure 6:**
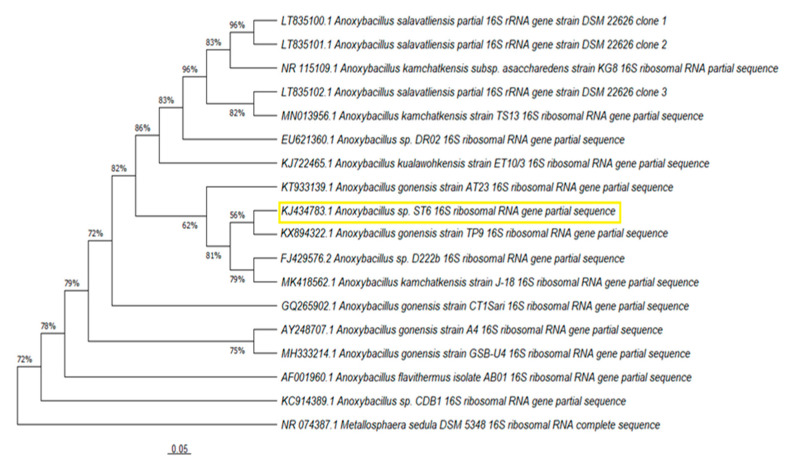
Phylogenetic tree depicting the relationships between strain ST6 and related taxa. The used outgroup was *Metallosphaera sedula* DSM 5348.

For 16S rRNA analysis, 1051 nucleotides were specified for ST3 and 1000 nucleotides for the ST6 strain. Following BLAST analysis, the sequences were submitted to GenBank. KJ434781 for strain ST3 and KJ434783 for strain ST6 were assigned as the accession numbers for the sequences. 16S rRNA analysis revealed that strains ST3 and ST6 were members of the genus *Anoxybacillus*. Figure 5 and Figure 6 present, respectively, the neighbor-joining-constructed phylogenetic trees for strain ST3 and strain ST6. It was determined that the partial 16S rRNA sequence of ST3 strain was 100% similar to *Anoxybacillus flavithermus* strain HBB-134 and *Anoxybacillus kestanbolensis* strain ACT14, respectively. It also indicated 99.90% homology to *Anoxybacillus flavithermus* strain 8POT11. The partial 16S rRNA sequence of strain ST6 showed 99.10%, 98.8%, and 98.7% homology to *Anoxybacillus gonensis* strain TP9, *Anoxybacillus gonensis* strain AT23, and *Anoxybacillus kamchatkensis* strain J-18, respectively.

### 3.3. Determination of Biodegradation by FTIR

**Figure 7:**
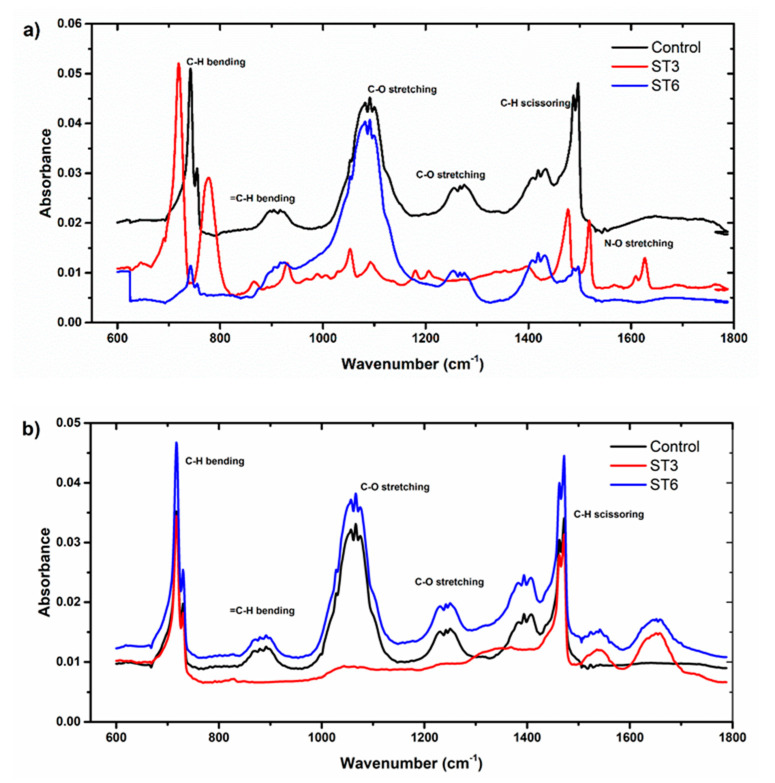
ATR-FTIR spectra in the region 600–1800 cm^−1^ of (**a**) 50 µm-sized and (**b**) 150 µm-sized PE-MPs inoculated with ST3 and ST6 strains.

With the inoculation of the thermophilic bacteria into PEs, several significant peaks at 720 and near 1450 cm^−1^ were assigned to the C-H alkyl bend, whereas the peak at 905 cm^−1^ was attributed to the =C-H (Figure 7a,b). The peaks between 1000, 1050, and 1200 cm^−1^ were for the C–O phenolic bands. In addition, the N-O peaks (1500–1600 cm^−1^) were mainly assigned.

Figure 7 shows the peak near 1475 and 1465 cm^−1^ due to the CH_2_ scissoring vibration which also shows a reduction of carbonyl bands [33]. The peaks at 1500 and 1600 cm^−1^ are attributed to the possible formation of amine groups [65]. Overall, the replacement of the carbonyl band with the amine band indicates that thermophilic bacteria form a biofilm layer on the polymer surface, proving that it causes a change in the chemical structure of PE [26]. With this information, it is easy to understand the degradation mechanisms of PEs.

To provide support to this claim, CI was determined to consider the degradation of the PE. CI is constantly used to assess the surface oxidation level of polymers, especially for polypropylene and polyethylene [66,67]. Carboxyl indices of 50 µm PE and 150 µm PE carboxyl indices for ST3 and ST6 were determined as 1.888, 0.748, 1.905, and 0.522, respectively (Table 3).

### 3.4. Morphology of Microplastics

**Figure 8:**
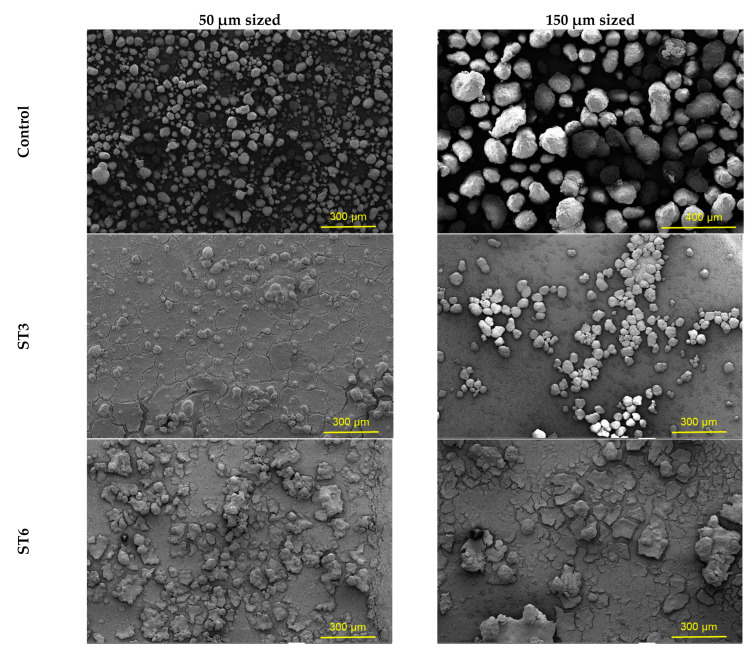
Surface changes of microbial degraded PE-MPs at magnifications of ×250.

Physical, chemical, and biological factors such as surface area, degrees of crystallinity, and molecular weight play an essential role to assess the degradation of polymer molecules [26,68]. SEM scans of raw and incubated PE-MPs revealed particles of various sizes and shapes, as shown in Figure 8. Having irregular folds in the polymer structure of raw polyethylene supports the formation of biofilms by adhesion of microorganisms to the surface.

## 4. Discussion

Enzymes secreted by thermophilic microorganisms have recently been of great importance in biotechnology [69]. One of these enzymes, alpha amylase, is widely used in biotechnology for many different purposes [70]. Both thermophilic microorganisms used in this study were good amylase producers. Therefore, they may be important from a biotechnological point of view.

The results revealed that particles with a diameter of 50 µm deteriorated significantly. In contrast to the findings of this study, polyethylene particles were known as less oxidized polymers, making them more robust than other polymers [67]. PE is relatively inert due to its hydrophobicity, and possible degradation of this polymer by microorganisms leads to losses of its properties including structural and mechanical deformations [71]. Until recently, only a few of the bacteroids including *Klebsiella*, *Micrococcus Staphylococcus*, *Pseudomonas,* and *Streptococcus* were identified as polymer degradation microbial species [72]. The recent studies on the biodegradation of MPs using different microbial degraders have increased [73]. For example, *Enterobacter* and *Pseudomonas* exhibited significant biodegradation of plastics, showing up to 15% weight loss in 120 days [74]. Similarly, Maroof et al. (2021) also revealed that a new bacterial strain, *B. siamensis*, showed a degradation ability with a percentage of 8.46% LDPE after 90 d of incubation [75].

However, the current analytical methods for the detection of polymer degradation seem very obscure due to the fact that the weight loss and surface structure changes resulting from the degradation of additives [28]. In this regard, the carbonyl index offers a great opportunity as a robust, reliable method to assess the biodegradability of polymers such as polyethylene and polypropylene. Although the carbonyl index has been widely used to observe the oxidation reactions that take place on the polymer, only a few studies, to the best of our knowledge, have looked at the carbonyl index of microbially decomposed microplastics. The determination of the carbonyl index makes it easier to measure and identify degradation level; hence, it shows a strong correlation with the average molecular weight of the polymers [76]. Dey et al. (2020) reported a 20-fold increase in the carbonyl index by investigating the catabolic repertoire of natural bacteria for plastic biodegradation in one of their studies [77]. Akarsu et al. (2022) also reported higher carbonyl indexes ranging from 0.23 to 0.53 [63]. Normally this means that ST3 and ST6 strains have better capability to degrade polyethylene compared to the microorganisms in their study.

Cracks and grooves were observed in this study as proof of the deterioration of the plastic beads because of the activity of the bacteria. Surface-level degradation was detected in higher 50 µm sized particles. Consistent with our findings, García-Depraect et al. (2022) also determined that a higher degradation level was detected at the lowest particle size tested [60].

Current research also revealed that the specific surface area of PE was higher than most polymers including polystyrene (PS) and polyvinyl chloride (PVC) [78,79]. However, dimensional alterations, abnormalities, cracks, and/or holes were reported for both bacteria and PE sizes, as regards the length of all samples. This finding is consistent with earlier studies. Curiously, authors demonstrate the extent of bacterial colonization and degradation using SEM micrographs of erosions, voids, and pores formed in plastic films [61]. Similarly, Sowmya et al. (2015) reported the occurrence of holes, scions, and cracks on the surface of the PE with fungal consortia [80]. Moreover, some researchers also investigated the bacteria surface interactions [63,81]. According to this, the secretion of extracellular enzymes by the isolates results in the formation of a biofilm on the cell surface. Consequently, enzymes entering the polymer pores weaken the polymer properties [82].

## 5. Conclusions

The environmental pollution caused by plastics poses a great threat to society, health, and the economy. The biodegradation potential of polyethylene can contribute to a better understanding of ways to promote the reduction of plastic and microplastic pollution. In this study, microplastics were efficiently used as a carbon source and degraded with two thermophilic bacteria. In addition, the relatively high levels of PE-MP degradation with alpha-amylase showed promising results.

FTIR and SEM analyses are the most used biodegradation detection methods. FTIR results indicated that the decomposition of PEs resulted in the formation of functional groups. Consequently, SEM analysis showed disturbances such as scions and holes.

The degradation performance of microplastics with various physical and chemical properties must still be further investigated in future studies.

## Figures and Tables

**Table 1:** Bacterial strains associated with microplastic degradation [27].

Genus-Strain	Source	MP Type ^1^	Duration Time (Day)	Biodegradation Analysis Method				References
				Weighting	FTIR	SEM	Other ^2^	
*Bacillus 27*	Mangrove sediment	PP	40	+	+	+	+	[37]
*Rhodococcus 36*	Mangrove sediment	PP	40					[37]
*Bacillus gottheilii*	Mangrove ecosystems	PE, PET, PP, PS	40	+	+	+	-	[33]
*Enterobacter asburiae YT1*	Plastic-eating waxworms	PE	28	+	+	+	+	[38]
*Bacillus YP1*	Plastic-eating waxworms	PE	28	+	+	+	+	[38]
*Bacillus subtilis MZA-75*	Soil samples	PUR	28	-	+	+	+	[39]
*Pseudomonas*	Digester sludge	PLA	40	-	-	+	+	[40]
*Bacillus MYK2*	Digester sludge	PLA	40	-	-	+	+	[40]
*Pirellulacease*	Fresh water	PE, PP	21	-	-	-	+	[41]
*Escherichia coli*	-	PU	3	+	-	+	+	[42]
*Alcanivorax borkumensis*	Floating plastics in oceans	PET, LDPE, PS	1–6	+	+	-	-	[43]
*Lysinibacillus* sp.	Plastic samples at surface water	PE	180	+	+	+	-	[44]
*Alcaligenes faecalis*	Municipal dumpsites	LDPE, HDPE, PES	15	+	+	+	-	[45]
*Bacillus cereus*	Municipal dumpsites	LDPE, HDPE, PES	15	+	+	+	-	[45]

^1^ LDPE: Low-density polyethylene; HDPE: High-density polyethylene; PES: Polyethersulfone; PET: Poly Ethylene Terephtalate; PLA: Polylactic acid; PS: Polystryene; PU: Polyurethane. ^2^ Other analysis methods include atomic force microscopy, gas chromatography-mass spectroscopy, and tensile strength.

**Table 2:** Differential phenotypical characteristics of the strains ST3 and ST6 (-: not secreted; +: moderate secreted; ++: good secreted; +++: excellent secreted).

Characterization	Strain ST3	Strain ST6
Motility	+	+
Gram	+	+
Spore	+	+
NaCl range (%, *w*/*v*)	1–7.5	1–7.5
NaCl optimum (%, *w*/*v*)	2.5–5	1–5
Temperature growth range	45–90	45–90
Optimum temperature (°C)	55–60	55–60
pH growth range	4.5–8.5	5.5–9.0
Optimum pH	5.5–6.0	6.0
Urease	-	+
β-galactosidase	+	+
Amylase	++	+++
Protease	-	+
Catalase	+	++
Oxidase	+	+
Citrate	+	+
Phosphate	+	+
Acid production from:		
-Glucose	+	+
-Lactose	+	+
-Sucrose	+	+

**Table 3:** CI values of raw and incubated PE-MPs.

Polyethylene	Raw	ST3	ST6
50 µm	0.709	1.888	1.905
150 µm	0.370	0.748	0.522

## Data Availability

The datasets used and/or analyzed during the current study are available from the corresponding author on reasonable request.
